# Omega-3 Docosahexaenoic Acid Is a Mediator of Fate-Decision of Adult Neural Stem Cells

**DOI:** 10.3390/ijms20174240

**Published:** 2019-08-30

**Authors:** Amanda Lo Van, Mayssa Hachem, Michel Lagarde, Nathalie Bernoud-Hubac

**Affiliations:** Univ-Lyon, Inserm UMR 1060, Inra UMR 1397, IMBL, INSA-Lyon, 69100 Villeurbanne, France

**Keywords:** omega-3 fatty acids, docosahexaenoic acid, neural stem cell, adult neurogenesis, neuroprotection

## Abstract

The mammalian brain is enriched with lipids that serve as energy catalyzers or secondary messengers of essential signaling pathways. Docosahexaenoic acid (DHA) is an omega-3 fatty acid synthesized de novo at low levels in humans, an endogenous supply from its precursors, and is mainly incorporated from nutrition, an exogeneous supply. Decreased levels of DHA have been reported in the brains of patients with neurodegenerative diseases. Preventing this decrease or supplementing the brain with DHA has been considered as a therapy for the DHA brain deficiency that could be linked with neuronal death or neurodegeneration. The mammalian brain has, however, a mechanism of compensation for loss of neurons in the brain: neurogenesis, the birth of neurons from neural stem cells. In adulthood, neurogenesis is still present, although at a slower rate and with low efficiency, where most of the newly born neurons die. Neural stem/progenitor cells (NSPCs) have been shown to require lipids for proper metabolism for proliferation maintenance and neurogenesis induction. Recent studies have focused on the effects of these essential lipids on the neurobiology of NSPCs. This review aimed to introduce the possible use of DHA to impact NSPC fate-decision as a therapy for neurodegenerative diseases.

## 1. Introduction

The human brain is a network of a great diversity of cells that ensure proper cerebral function. Neurodevelopment during embryogenesis is particularly important. It was thought for a long time that neurons, the main actors of cerebral electrical activity, were only produced during neurodevelopment and that their number was definite at the end of brain maturation. While still debated, the process of the creation of new neurons has been observed more and more in the adult human brain [1,2,3,4,5]. Adult neurogenesis is made possible by the maintenance of a pool of pluripotent cells, neural stem/progenitor cells (NSPCs), and their subsequent differentiation into mature functional cells, namely neuronal cells for neurogenesis [6,7,8,9]. There is, however, a loss of neurogenesis rate with age [10], which could explain the still ongoing debate around the existence of adult neurogenesis.

Docosahexaenoic acid (DHA), an omega-3 polyunsaturated fatty acid, is uniquely enriched in the brain and the retina [11,12], and is required for proper human brain development and visual functions [13,14,15,16]. Several studies conducted both in animal models and in humans have suggested that an adequate dietary intake of omega-3 fatty acids can prevent cognitive decline and attenuate the physiological disturbances of the brain that are associated with ageing or with neurological disorders, such as Alzheimer’s disease (AD) and Parkinson’s disease (PD) [17,18,19,20,21]. This is particularly interesting and could be linked to the important role played by DHA in NSPC metabolism. With descriptions of adult neurogenesis and DHA metabolism, we have emphasized the importance of DHA for NSPC cell fate decision-making, and updated the current knowledge of DHA and omega-3 fatty acid effects on neurogenesis and neuroprotection.

## 2. Adult Neurogenesis Is Linked with a Metabolic Shift

### 2.1. Adult NSPC Origin and Neurogenic Niches

Mammalian brains are composed of a diversity of cell types that ranges from glial cells (i.e., astrocytes, microglia, and oligodendrocytes) to neuronal cells (i.e., neurons). During embryonic development, all cells are generated from a single cell type: the embryonic neural stem progenitor cells. These are pluripotent cells that can divide symmetrically to increase the cell population pool, or proliferate—divide asymmetrically to produce fate-committed cells, a process of differentiation or specification [22,23,24,25]. Rapid proliferation followed by differentiation into glial cells, gliogenesis, or neuronal cells, neurogenesis, has been observed in mice embryos [26,27,28,29]. This ensures proper brain development for cognitive functions. In rodent neocortex, neurogenesis mainly occurs from E12 to E18, followed by gliogenesis starting from E18 up to postnatal age. Oligodendrogenesis happens in several waves during embryogenesis and postnatally. It is generally accepted that NSPCs that have divided less are more likely to produce neurons compared to NSPCs that have undergone more divisions, which are considered more gliogenic [30].

However, two neurogenic niches are found in mouse brain: the subgranular zone (SGZ) of the dentate gyrus of the hippocampus [8,31,32] and the subventricular zone of the lateral ventricles (SVZ) [6,33,34,35]. Some research groups suggest that adult NSPCs originate from embryonic NSPCs that went into a quiescent state for maintenance of a life-long pool [36,37,38,39,40]. Adult NSPCs from the two niches both have the same capacity to differentiate into neuronal or glial cells [6,7,8,9], although the functions they serve are suggested to be different depending on the production site and their migration trajectory. The SVZ NSPCs have been mainly observed to integrate into the olfactory bulb and to serve the functions of olfactory recognition and memory [41,42], while the SGZ NSPCs are mainly involved in hippocampal functions, including learning and spatial memory and mood [43,44,45].

In adult age, neurogenesis is significantly lower than in young individuals [10]. The determining factors for the cell-fate decision of adult NSPCs are still under study, but most studies have converged to the hypothesis that low adult neurogenesis is observed because most NSPCs are dormant/quiescent [46], and that exiting this stage to proliferate or differentiate does not lead to the birth of healthy and functional cells, rather leading to the premature death of the cells [47,48,49,50]. This phenomenon has also been suggested to be involved in neurodegenerative diseases [51]. It is thus important to identify the signaling pathway behind adult NSPCs’ complex metabolism. We note that human adult neurogenesis is still an open debate, although evidence of the existence of neural stem cells has been observed in human brains [1,2,3,4,5]. Numerous reviews have detailed the biology of adult NSPCs [52,53,54,55,56,57,58].

### 2.2. Cell-fate Decision and Increased Lipogenesis

The factors inducing the transitions between proliferation and differentiation and between neurogenesis and gliogenesis are still not fully understood. They include extrinsic factors: cytokines, growth factors, neurotransmitters, and morphogens. Intrinsic factors are also involved, and include transcription factors, epigenetic regulators, and non-coding RNAs such as microRNAs [59,60,61]. They have been covered extensively in previous reviews [56,58,62,63,64]. Proliferation and differentiation of NSPCs is energy-consuming, and cells need substrates to initiate cell cycle entry and progression [65]. Possible stocks include glucose and lipids from the cell membrane, as well as uptake from the extracellular environment. Proliferating stem cells favor high glycolysis for energy production and proliferate in hypoxic conditions with low oxygen consumption, as observed in other stem cells [66,67]. This has also been observed in quiescent stem cells with less glycolysis and even less oxygen consumption. However, a metabolic shift has been observed during fate commitment of the stem cells, ensuring differentiation and maturation. The energy production site of stem cells is the mitochondria. Mitochondria use oxidative phosphorylation (OXPHOS) to produce ATP (adenosine triphosphate), which has been shown to be required to initiate differentiation [68,69]. As a result, there is an elevation in reactive oxygen species (ROS) during neurogenesis [70]. ROS might act on differentiating pathways [71] but have a downfall effect. ROS elevation intracellularly is linked with oxidative stress and DNA damage [72,73,74], and could lead to the premature death of newly differentiated cells [75,76,77]. The essential role of the mitochondria has been detailed in previous reviews [78,79,80].

**Figure 1:**
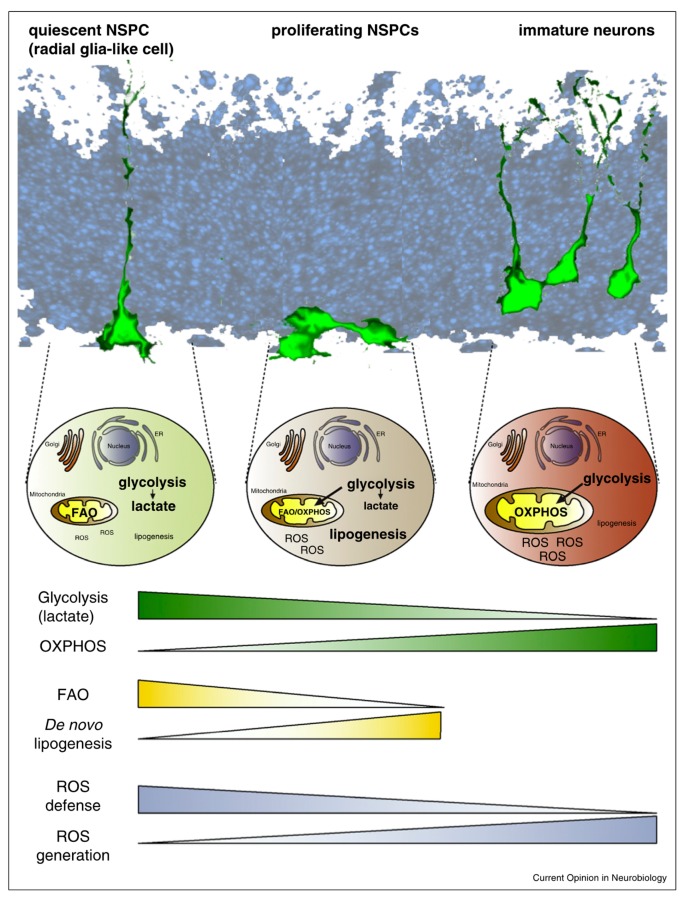
Representation of the major metabolic pathway changes of glycolysis, oxidative phosphorylation (OXPHOS), fatty acid oxidation (FAO), lipogenesis, and reactive oxygen species (ROS) generation and defense in neural stem/progenitor cells (NSPCs) during cell-fate decision. Illustrated for NSPC metabolism from quiescent cells to immature neuron differentiation in the adult subgranular zone (SGZ) and the developing forebrain. From Knobloch et al. [86].

NSPC metabolism has also been shown to depend on lipid biogenesis and metabolism. One research group showed that impairment of lipogenesis, the production of lipids from end-products of glucose metabolism, could prevent NSPC differentiation [81]. They also discovered that fatty acid oxidation (FAO) inhibition in SGZ NPSCs led to quiescent cell death and reduction of cell proliferation [82]. Another group found that FAO blocking decreased proliferation of SVZ NSPCs [83]. Both discoveries suggest that FAO is required for sustaining energy production in proliferating cells, while lipogenesis is necessary for differentiation. Interestingly, providing fatty acids as a source of energy instead of glucose increased the developmental stage of human-induced pluripotent stem cell-derived cardiomyocytes, with a preferential switch to energy production via FAO [84]. In another model of human pluripotent stem cells, researchers observed that lipid deprivation maintained cells in a pluripotent state [85]. FAO occurs in mitochondria, while de novo lipogenesis takes place in the cytoplasm, emphasizing once more the close link between mitochondrial function and NSPC metabolism [86] (see summary in Figure 1). Lipids are stored in cell bilayer membranes, in structures of higher complexity such as glycerophospholipids. They serve many purposes, like the above-mentioned energy production, but are also key players of signaling pathways for inflammation, oxidation, or apoptotic cascades. Among the abundant lipids composing brain cells, there is evidence of an enrichment in the omega-3 fatty acid DHA in the brain and the retina compared to other tissues [11,12], and poor DHA and other omega-3 fatty acid dietary supply during pregnancy leads to high risks of poor visual and cerebral development of infants and to increased risk of cognitive function impairments and possibly neurodegenerative diseases (as reviewed previously [16,87,88,89,90]). This hints at the important role played by DHA and other omega-3 fatty acids in NSPC metabolism.

## 3. DHA Is a Major Component of Brain Lipids

### 3.1. Omega-3 DHA Levels in the Brain

Omega-3 fatty acids are polyunsaturated fatty acids that are characterized by the presence of double bonds, the first double bond being present at the carbon 3, counting opposite to the terminal methyl group. They are essential fatty acids that are synthesized at low levels *de novo* in human metabolism and are mainly incorporated through our diets. The main omega-3 fatty acids are, in increasing chain length order: alpha-linoleic acid (ALA,18:3n-3), eicosapentaenoic acid (EPA,20:5n-3), and DHA (22:6n-3). Omega-3 fatty acids are particularly enriched in oily fishes such as salmon, anchovies, or sardines for DHA dietary supply, but also in vegetable oils (i.e., soybeans) for ALA supply [91]. DHA synthesis is possible via the elongation-desaturation pathway from ALA through conversion into EPA. Although this DHA supplementation is considered to be sufficient for proper brain DHA accretion [92], it still requires the dietary supplementation of ALA.

DHA is seldom observed in the free fatty acid form in brain cells; it is mostly incorporated into the glycerophospholipids contained in the cell membranes. The main phospholipids are phosphatidylcholines (PC), phosphatidylethanolamines (PE), phosphatidylinositols (PI), and phosphatidylserines (PS), with a preferential abundance in ethanolamine glycerophospholipids (EtnGpl) [93,94,95]. In aged mammalian brain, DHA is recovered mainly in the cortex and cerebellum [95]. An exhaustive study of determination of the fatty acid composition in several brain regions according to experimental models of mice and different diets was performed and showed that DHA content is very dependent on dietary supply [96].

### 3.2. DHA Metabolism and Oxygenated Metabolites

There is competition for enzymes and incorporation into phospholipids between omega-3 and omega-6 fatty acids, which are polyunsaturated fatty acids with double bonds starting from the carbon 6, away from the terminal methyl group. Moreover, omega-6 signaling cascades include the formation of cytokines, some of which are key players of inflammatory pathways, through synthesis involving the same enzymes as those required by omega-3-derived metabolite generation [97]. Being highly unsaturated, DHA has the potential to be oxygenated by various lipoxygenases to produce oxylipins that regulate several biological processes within the brain. Although being identified only at low levels in vivo in the brain, these mediators are mainly produced by lipoxygenase action and include hydroxylated DHA, while some of them can be metabolized into potent mediators, such as protectin D1 (PD1), resolvins, and maresins [98,99]. One isomer of PD1 was identified by our group and named protectin DX (PDX [100]), and was observed in mice brains in vivo [101]. Other reviews have explored in more details the different derivatives of omega-3 fatty acids and their potent effects as neuroprotective agents or neurogenesis inducers [102,103,104]. As mentioned before, fatty acids also participate in cell energy production by serving as substrates for FAO.

### 3.3. DHA Delivery Strategy for Better Brain Accretion

For therapeutic use, the primary step is to ensure proper DHA delivery to the brain from blood through the blood–brain barrier (BBB). We previously published a summary of the known mechanisms of DHA brain accretion [105]. BBB is a physiological barrier preventing the invasion of toxic compounds and ensuring correct homeostasis for the healthiest brain conditions. Nutrients from diet are transported in the blood stream up to the BBB. DHA is either bound to albumin in its free form and within lysophosphatidylcholine (LysoPC) [106], or is taken up by lipoproteins [107]. The mechanisms of entry into endothelial cells are still under debate and may include passive diffusion of cleaved DHA or LysoPC–DHA from albumin and lipoproteins [108], and active transportation via specific membrane proteins. An emerging candidate is the major facilitator superfamily domain-containing protein 2A (Mfsd2a), which has been proven to preferentially bind LysoPC–DHA compared to the free fatty acid form [109,110]. Mfsd2a-knockout animal models showed decreased DHA brain levels [109], microencephaly [111], and dysregulated lipogenesis and phospholipid accretion in cell membranes [112]. Other studies showed that LysoPC–DHA supplementation through diet increased brain DHA content as compared to free fatty acid DHA [113,114], in particular in the cerebellum, hippocampus, striatum, and amygdala, with improvement of memory functions [115]. One group recently showed that DHA esterified in PE or PC was better taken up by the brain compared to DHA in triacylglycerols in adult rats [116], as was shown previously [117].

**Figure 2:**
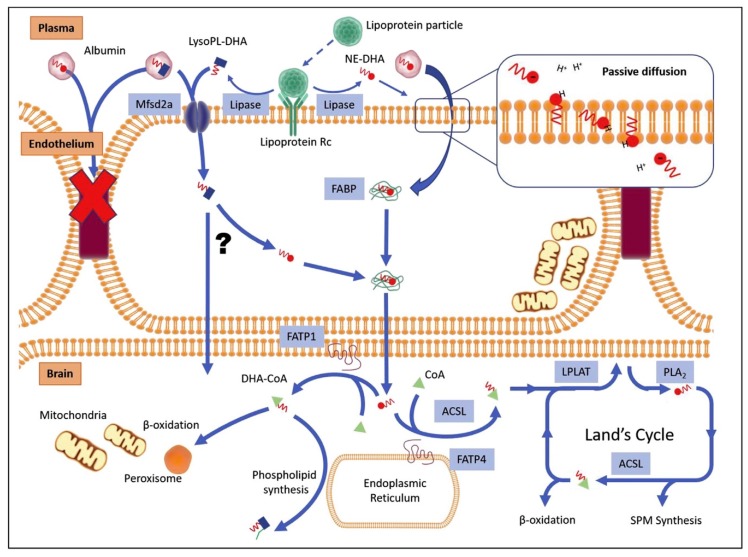
Docosahexaenoic acid (DHA) uptake through the blood–brain barrier (BBB) and metabolism in brain cells. Transfer from plasma to endothelial cells is possible through passive diffusion and active transport of non-esterified DHA (NE-DHA) or lysophospholipid containing DHA (LysoPL), including LysoPC–DHA. Active transport involves lipoproteins and Mfsd2a. Inside endothelial cells, NE-DHA and DHA cleaved from LysoPL–DHA are bound to fatty acid binding proteins (FABP) to cross the intercellular space to reach brain cells. DHA then participates in cell metabolism for energy production of CoA and production of signaling mediators, specialized pro-resolving mediators (SPM), producing beta-oxidation. For further details, refer to original figure from Lacombe et al. [129].

We showed previously that a synthetic analog of LysoPC–DHA, called AceDoPC^®^ for 1-acetyl,2-docosahexaenoyl-PC [118,119,120], was better incorporated into the brain of rats injected intravenously with AceDoPC compared to the free fatty acid form of DHA and PC–DHA [121]. Preliminary results showed that DHA was particularly accumulated in specific brain regions that included the hippocampus, one neurogenic niche of mammalian brains. Moreover, we also observed that this preferred vector of DHA to the brain was neurogenic on adult NPSCs in a model of hypoxia in vitro [122], and thus could serve two purposes: increasing DHA accretion and providing neuroprotection. DHA and other fatty acid transport intracellularly is also of primary importance, and is mainly handled by fatty acid binding proteins [123,124]. Several studies have highlighted the importance of these proteins for brain development [125,126]. DHA, and phospholipids containing DHA, also have the ability to modulate membrane composition through lipid rafts and act on the trafficking of signaling molecules, which could, in turn, impact neuronal processes [127,128]. A comprehensive review of DHA brain uptake and metabolism was published recently [129] (see Figure 2).

## 4. DHA Modulation of Neuroprotection and Neurogenesis for Therapeutics

### 4.1. DHA Impacts Cell-Fate Decision and Survival of Newly Born Cells

Much work is now ongoing to determine the exact mechanisms underlying the possible use of DHA and other omega-3 fatty acids to influence the fate and behavior of neural stem cells. As mentioned above, DHA can serve as a substrate for the production of energy for the progression of cell cycle and cell division. To our knowledge, only a few studies have focused on the effects of DHA on the proliferation of adult NSPCs, compared to numerous studies on embryonic NSPCs as well as studies on cancer cells. We report here the work of Sakayori et al., who showed that arachidonic acid (ARA, an omega-6 fatty acid) and DHA have distinct effects on the metabolism of NSPCs [130]. DHA supplementation to adult NSPCs from rats showed increased proliferation, as did ARA supplementation. However, in a medium deprived of growth factors, DHA induced neurogenesis while ARA increased astrogliogenesis. They observed that high concentrations of DHA (10 micromolar and above) could be detrimental to the proliferation capacity of the NSPCs, which was also observed by our group [122], and which interestingly was not observed when DHA as esterified within AceDoPC^®^, a DHA-containing phospholipid. These high concentrations of DHA have also been studied in cancer cells, and they were shown to reduce proliferation, invasion, and also survival of the cells [131,132], due to the accumulation of ROS intracellularly and damage to the mitochondria that led to the activation of the apoptotic pathway [133].

One emerging hypothesis is that DHA induces the exit of the cells from the S-phase of the cell cycle to initiate differentiation into specialized mature cells [134]. If the right balance is found between the non-detrimental effects of DHA, this could lead to an enrichment in mature cells from NSPCs, as well as maintained cell proliferation. Transgenic mice expressing the *Caenorhabditis elegans fat-1* gene are able to convert omega-3 fatty acids from omega-6 fatty acids, leading to an abundance of the former lipids. These mice showed an enhancement of proliferating cells in the SGZ of young adult mice and an increase in performance in spatial learning tests [135], which the authors explained as an increase in neurogenesis that in turn helped with hippocampal memory. Numerous reviews have reported the effects of DHA on the neurogenesis of neural stem cells, mainly embryonic [136,137,138,139]. DHA promotes neurogenesis in vitro but does not promote gliogenesis [130,140,141,142]. DHA can act on transcription factors such as Hes1 (hairy and enhancer of split 1, a basic helix–loop–helix transcription factor), increasing p27kip1 level, a cyclin-dependent kinase inhibitor, thus stopping cell cycle progression [143] and inducing differentiation [144,145,146]. NeuroD (helix–loop–helix transcription factor) and MAP2 (microtubule-associated protein 2) levels were also increased with DHA addition, suggesting that protein kinase C-dependent mechanisms might be involved. Another candidate pathway is the activation of G-protein coupled receptor 40 [140]. The increased number of neurons derived from NSPCs could also be due to a combination between an increased number of NSPCs differentiating into neurons and a pro-survival effect on newly born neurons. DHA and omega-3 fatty acids enhance neuroprotection by anti-apoptotic [143,147,148], anti-oxidative [149,150,151,152], and anti-inflammatory effects [153,154]. Furthermore, as mentioned above, DHA metabolites also present pro-survival activity.

### 4.2. DHA Induces Neuroprotection and Increased Neurogenesis

Normal aging is marked by neurogenesis loss, and thus could be impacted by DHA supply. However, most studies have indicated that DHA effects on neurogenesis were mostly relevant when compared with subjects fed with DHA-deficient diets [155,156], except for the recent study mentioned previously with DHA supplementation through LysoPC–DHA [115]. This could hint at the hypothesis that DHA effects could be more relevant in patients with impaired neurogenesis and cognitive functions rather than healthy patients, and that the form of DHA supply is particularly important. Therefore, DHA has been studied as a potent therapy to treat neurodegeneration.

These studies are, however, limited, as there is no common agreement on the best animal models with which to study major neurodegenerative diseases such as AD or PD. The most studied pathology is AD, and our group previously published a review of the effects of DHA on this widespread neurodegenerative disease [157]. It was recently published that AD patients have impaired adult hippocampal neurogenesis compared to healthy patients [5], as was hypothesized before [158]. DHA exerts neuroprotective effects mainly by impairing beta-amyloid production [159,160]. Few of these studies showed enhancement of memory performances, and only one reported higher proliferation of cells in the SGZ with the administration of 2-hydroxy-DHA [161]. One explanation could be that the models of AD used did not completely recapitulate the disease symptoms and did not impair neurogenesis; however, this recent review hinted at the use of a model of AD with disturbed lipid metabolism in the SVZ [162]. In PD animal models, DHA has proven to be neuroprotective [163,164,165,166], but there are no reports of increased neurogenesis.

As for acute brain injuries, two recent studies were published on DHA effects upon traumatic brain injury (TBI) [167,168]. Of particular interest, one group transplanted NSPCs following TBI, with DHA or without, and observed an increase of neurogenesis with DHA supplementation compared to NSPCs transplanted alone in the SVZ [167]. Stroke induces important brain lesions, and improvement of recovery is of particular interest. Numerous studies have been published on DHA supply post-stroke, and some showed increased neurogenesis in the cortex and striatum with DHA complexed with albumin [169], increased neurogenesis and oligodendrogenesis in the striatum with an omega-3 diet [170], and improvement of neurobehavioral scores [21]. We also observed increased proliferation with DHA supply to NSPCs after oxygen and glucose deprivation, a protocol mimicking hypoxia–ischemia, and we noted an increase in neurogenesis, this effect being higher when DHA was esterified in AceDoPC^®^ [122]. However, only a few studies have focused on the capacity of NSPCs to regenerate the brain, and particular interest lies in the study of the neurogenic niches that are the SVZ and SGZ of the hippocampus.

## 5. Conclusions

This review has summed up the current knowledge of the effects of omega-3 fatty acids on adult NSPCs, with a particular focus on DHA. Omega-3 fatty acids can be incorporated into the cell membrane in different forms, but they have first to cross the BBB in order to reach the brain and the currently known neurogenic niches. They are actors and coordinators of the metabolic pathways involved in NSPC dynamics, and could be applied for therapeutics as prevention or cure to neurodegenerative diseases, via better survival of the newly born cells from the remaining pool, a neuroprotective effect, and the induction of neurogenesis in spite of astrogliogenesis. Delivery strategies for better brain accretion of DHA are currently being tested in animal models and are necessary for enhancement of the putative neurogenic and neuroprotective effects. To bypass the effect of ROS accumulation and the risk of DHA toxicity, it is also suggested to focus future work on the effects of potent derivatives of omega-3, such as resolvins and protectins. We propose that further interdisciplinary studies which combine the understanding of NSPC fate decision and fate commitment with the comprehension of omega-3 fatty acid biogenesis and downstream signaling, are of uttermost interest for the development of a relevant, putative, and non-invasive therapy against neurodegeneration and towards brain regeneration therapy.

## Abbreviations

DHA	Docosahexaenoic acid
NSPC	Neural stem/progenitor cells
AD	Alzheimer’s disease
PD	Parkinson’s disease
SGZ	Subgranular zone
SVZ	Subventricular zone
OXPHOS	Oxidative phosphorylation
ROS	Reactive oxygen species+
ATP	Adenosine triphosphate
FAO	Fatty acid oxidation
ALA	Alpha-linoleic acid
EPA	Eicosapentaenoic acid
PC	Phosphatidylcholine
PE	Phosphatidylethanolamine
PI	Phosphatidylinositol
PS	Phosphatidylserine
EtnGpl	Ethanolamine glycerophospholipid
PD1	Protectin D1
PDX	Protectin DX
BBB	Blood–brain barrier
LysoPC	Lysophosphatidylcholine
LysoPC-DHA	Lysophosphatidylcholine-DHA
Mfsd2a	Major facilitator superfamily domain-containing protein 2A
AceDoPC^®^	1-acetyl,2-docosahexaenoyl-PC
PC-DHA	Phosphatidylcholine-DHA
NE-DHA	Non-esterified DHA
LysoPL	Lysophospholipid
SPM	Specialized pro-resolving mediators
ARA	Arachidonic acid
Hes1	Hairy and enhancer of split 1
MAP2	Microtubule-associated protein 2
TBI	Traumatic brain injury
